# Effects of flexor reflex stimulation on gait aspects in stroke patients: randomized clinical trial

**DOI:** 10.1186/s12984-024-01377-y

**Published:** 2024-05-28

**Authors:** Aida Sehle, Christian Salzmann, Joachim Liepert

**Affiliations:** 1 Kliniken Schmieder, Allensbach, Germany; 2 Lurija Institute, Allensbach, Germany

**Keywords:** Neurorehabilitation, Gait rehabilitation, Stroke, Flexor reflex, Gait analysis

## Abstract

**Background:**

Gait deficits are very common after stroke and therefore an important aspect in poststroke rehabilitation. A currently little used method in gait rehabilitation after stroke is the activation of the flexor reflex (FR) by electrical stimulation of the sole of foot while walking. The aim of this study was to investigate the effect of FR stimulation on gait performance and gait parameters in participants with stroke within a single session of flexor reflex stimulation using Incedo™.

**Methods:**

Twenty-five participants with subacute (n = 14) and chronic (n = 11) stroke were enrolled in the study. Motor functions were tested with a 10-m walk test (10mWT), a 2-min walk test (2minWT), and a gait analysis. These tests were performed with and without Incedo™ within a single session in randomized order.

**Results:**

In the 10mWT, a significant difference was found between walking with Incedo™ (15.0 ± 8.5 s) versus without Incedo™ (17.0 ± 11.4 s, p = 0.01). Similarly, the 2minWT showed a significant improvement with Incedo™ use (90.0 ± 36.4 m) compared to without Incedo™ (86.3 ± 36.8 m, p = 0.03). These results indicate that while the improvements are statistically significant, they are modest and should be considered in the context of their clinical relevance. The gait parameters remained unchanged except for the step length. A subgroup analysis indicated that participants with subacute and chronic stroke responded similarly to the stimulation. There was a correlation between the degree of response to electrostimulation while walking and degree of improvement in 2minWT (*r* = 0.50, p = 0.01).

**Conclusions:**

This study is the first to examine FR activation effects in chronic stroke patients and suggests that stimulation effects are independent of the time since stroke. A larger controlled clinical trial is warranted that addresses issues as the necessary number of therapeutical sessions and for how long stimulation-induced improvements outlast the treatment period.

*Trial registration:* The trial was retrospectively registered in German Clinical Trials Register. Clinical trial registration number: DRKS00021457. Date of registration: 29 June 2020.

**Supplementary Information:**

The online version contains supplementary material available at 10.1186/s12984-024-01377-y.

## Background

The most common pattern of walking impairment poststroke is hemiparetic gait [1]. Hemiparetic gait is typically characterized by specific spatiotemporal patterns, including decreased cadence, prolonged swing duration on the paretic side, extended stance duration on the nonparetic side, and step length asymmetry when compared with the gait parameters of healthy subjects [2, 3].

Hemiparesis stands out as very frequent and widely recognized impairment that had been reported in approximately 65% of patients [4]. Gait recovery is an important aspect of stroke rehabilitation and a primary goal for most patients [5–7], because gait is of fundamental importance in the activities of daily living [8]. Thus, for improvement of poststroke gait impairment, numerous rehabilitative training methods have been developed, such as overground walking, cycling, treadmill walking, balance and cardiorespiratory training, repetitive standing exercise, proprioceptive neuromuscular facilitation, repetitive transcranial magnetic stimulation and electrostimulation [9–18].

Stimulating the flexor reflex (FR) through electrical stimulation of the sole of the foot has been proposed as a modality for gait rehabilitation after stroke, albeit with limited scientific evidence supporting its effects [19–22]. To the best of our knowledge, four studies have investigated the impact of FR stimulation during gait training in stroke patients. Among these, three studies delved into the effects of electrical FR stimulation during gait training in subacute stroke patients, revealing positive outcomes concerning walking speed [19–21] and gait parameters [19, 21]. Additionally, a recently published case report demonstrated favorable effects of FR stimulation during gait training, specifically in terms of walking speed and stride length, in a severely affected chronic stroke patient [22]. Furthermore, there is a gap in the existing literature regarding studies examining the effects of FR stimulation on spatio-temporal parameters of walking in poststroke patients (both subacute and chronic) within a single session.

Several studies have explored the impact of FR activation during walking in hemiplegic patients, focusing on technical and methodological aspects [23–25]. These aspects include electrode placement [23–25], variation in stimulation onset times [25], and parameters like stimulation frequency (Hertz) and pulse duration (milliseconds) [23].

The aim of this study was to examine the effect of FR stimulation during walking in participants with subacute and chronic stroke within a single session. Two hypotheses were tested:

- The gait performance (10-m walk test and 2-min walk test) is expected to show improvement within a single session of FR stimulation using Incedo™.
- Specific gait parameters (e.g., duration of the stance and swing phase, stride length, step height, and circumduction) are anticipated to be modified within a single session of FR stimulation using Incedo™.

## Methods

### Subjects

**Fig. 1:**
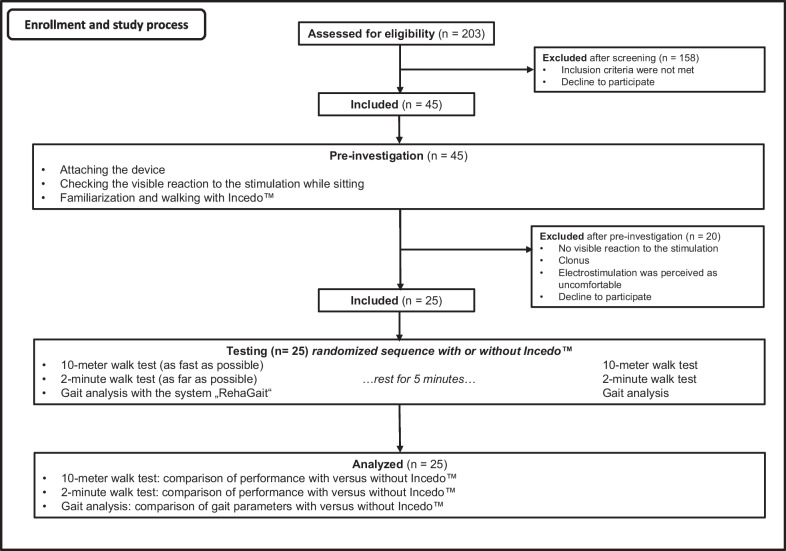
Flow diagram of the study

Overall 203 subacute (< 6 months after stroke) and chronic stroke (≥ 6 months after stroke) patients were identified as potential participants in this study from January 2020 to May 2022. Figure 1 shows the selection process in the study and the study process.

Inclusion criteria were unilateral lower extremity paresis resulting from a stroke (ischemic or hemorrhagic), an age of at least 18 years, ability to understand the instructions and to give informed consent for the participation in the study. Furthermore, patients had to be able to stand freely and walk without assistance and orthoses for at least 10 m, basically corresponding to the Functional Ambulation Category 3. While patients were expected to ambulate without the aid of a therapist or orthosis, the use of assistive devices such as a cane or walker was permitted during the testing phase to ensure safety and accommodate individual needs. Exclusion criteria were prior history of neurological illnesses or psychiatric conditions, lack of compliance, epilepsy, patients with heart pacemakers, severe heart or lung diseases, cancer, pregnancy, and skin lesions in the area where the electrode is positioned. Both inpatients and former patients from the department of Neurorehabilitation (Kliniken Schmieder Allensbach, Germany) were recruited to participate in this study.

After screening inclusion and exclusion criteria, 45 patients participated in a pre-investigation where the Incedo™ system, developed by Nordic NeuroSTIM ApS in Aalborg, Denmark, was presented and tested. This system is designed to enhance the natural gait by timely activation of the FR through precise electrical stimulation. It includes an impulse generator, electrodes, and a pressure-sensitive foot sensor integrated within the patient's shoe.

As soon as the sensor detects the foot lifting at the start of the swing phase, it sends a signal to the impulse generator. The generator then delivers a controlled electrical pulse specifically configured as four pulse trains, each consisting of five pulses at a frequency of 200 Hz, with each pulse having a duration of 1 ms, under a balanced biphasic pulse configuration. The electrodes, attached to the sole of the patient’s foot, facilitate this process.

The total duration for each stimulation sequence is always fixed at 230 ms, which is significantly shorter than the duration of the complete swing phase to ensure that the stimulation merely triggers the necessary reflex to assist the movement of the hip, knee, and ankle joints, rather than sustaining movement throughout the swing phase.

The Incedo™ device operates with the following specifications:

- Pulse: Balanced biphasic.
- Pulse Pattern: Four 15 Hz pulse trains consisting of 5 pulses each, delivered at 200 Hz.
- Pulse Duration: 1 ms.
- Intensity: Adjustable from 0 to 50 mA.
- Maximal Voltage: 270 V.
- Maximal Load: 5400 Ω.

The Incedo™ device is current controlled, ensuring precise and consistent delivery of stimulation. The foot sensor is adept at detecting changes in foot pressure, critical for the accurate timing of the stimulation. Notably, there is no delay from when the foot is lifted off the ground until stimulation is given. However, the FR that assists in leg movement does include an inherent delay of approximately 140 ms, carefully calculated to align with the most beneficial timing relative to the ongoing gait cycle.

The calibration of the Incedo™ device was conducted with the patient in a seated position. The stimulation intensity was gradually increased in increments of 0.5 mA, either until the patients exhibited a very good response or until the stimulation was perceived as uncomfortable. Prior to stimulation, patients were informed to communicate any discomfort during the process. Subsequently, patients had the opportunity to experience walking with the Incedo™ device. During walking, the stimulation intensity was readjusted. In this case, the stimulation during walking was gradually increased from the initial seated stimulation until it was perceived as uncomfortable by the patient. Following this, the stimulation was then systematically reduced by 0.5 mA until the patient could tolerate the stimulation comfortably. For participation in the study it was required that [1] a visible FR is triggered in the affected leg while sitting, [2] the patient tolerated the electrostimulation well, and [3] the patient can walk safely without a foot drop orthosis. If these criteria were met, the patient had enough time to get used to the device while walking. The patients walked with Incedo™ for up to 30 min.

After this pre-investigation 25 stroke patients were included in the study (Table 1). The other 20 patients had to be withdrawn. The withdrawal of the other 20 patients occurred due to various reasons: seven patients showed no visible reaction to the stimulation, two patients experienced clonus triggered in the affected leg by the electrostimulation, five patients found the electrostimulation uncomfortable, and six patients either did not want or were unable to participate in the study due to factors such as transitioning to a different rehabilitation phase in another location.

**Table 1:** Demographic and clinical data

Category	Results
Gender (male/female)	16/9
Age (years)^a^	62.8 ± 13.1
Phase (subacute/chronic)	14/11
Affected side (right/left)	9/16
Ischemic stroke / hemorrhage	21/4
Time since incident in participants with subacute stroke (weeks)^a^	9.2 ± 4.5
Time since incident in participants with chronic stroke (weeks)^a^	150.6 ± 113.2
Barthel Index (points)^a^	66,6 ± 19.4
Foot drop orthosis in everyday living (yes/no)	17/8

^a^Values are presented as mean ± 1 standard deviation

### Experimental procedure

Motor functions were tested with a 10-meter walk test (10mWT), a 2-minute walk test (2minWT), and a gait analysis. These tests were performed with and without Incedo^TM^ in randomized order. The randomization was done using a coin. A research assistant (C.S.) generated the allocation sequence, one of two research assistants (C.S. or A.S.) enrolled participants, and one of two research assistants (C.S. or A.S.) assigned participants to interventions. Patients had a five-minute break between these two examinations.

The 10mWT has been shown to be reliable and valid [26]. The performance is closely correlated with measures of strength, balance, and physical activity [26]. For 10mWT, the individuals were asked to walk 10 m ‘as fast as possible’. Performance time was measured in seconds.

The 2minWT is a popular and well-established walking test to obtain a detailed impression of walking ability [27]. However, the gold standard of endurance testing is considered to be the 6-min walk test [28]. Since some patients participating in this study were unable to walk for longer than two minutes we selected the 2minWT. In addition, the 2minWT can be well compared to the 6-min walk test [27, 29–31]. The distance walked in two minutes correlates well with the 6-min walking distances [30]. Particularly, the 2minWT is probably best for documenting the patient’s self-selected walking speed, because it minimizes fatigue effects [30]. For 2minWT, participants were requested to walk as far as possible for two minutes on a 30-m-long course. The distance they covered was measured in meters.

In addition, the threshold value and the stimulation stimulus intensity of Incedo™ during the measuments were recorded in the study protocol. The perception of the electrical stimulus was defined as the threshold value. Furthermore, the reaction to the FR stimulation was visually assessed by the examiner. The four-level classification was as follows: 'no response' indicates the absence of any visible muscle contractions during FR stimulation; 'slight response' signifies that FR stimulation induces a visible muscle contraction in the affected leg with minimal movement; 'moderate response' indicates that FR stimulation triggers movement in the affected leg, though not reaching the full range of motion; and 'good response' means that FR stimulation elicits a complete movement in the affected leg. These criteria were used to categorize and define the observed responses during both sitting and walking assessments. It was also noted whether patients walked with or without devices such as a cane during the walking tests. Moreover, it was recorded whether patients were wearing an orthosis in everyday life.

All patients underwent a gait analysis (RehaGait system, HASOMED GmbH, Magdeburg, Germany) over a distance of 30 m. The equipment includes two inertial sensors, which were mounted on the dorsum of the foot. The inertial sensors contain tri-axial accelerometers for measuring acceleration and tri-axial gyroscopes for measuring the angular velocity. Two sets of gait parameters were obtained for each patient: spatiotemporal (stride duration, cadence, stance phase duration, swing phase duration, single support duration, double support duration) and kinematic gait parameters (stride length, heel strike angle, toe off angle, maximum foot height and maximum circumduction). Heel strike angle is defined as the angle between the foot and the ground at the moment the heel makes contact to initiate the stance phase of the gait cycle. Toe off angle refers to the angle between the foot and the ground at the moment the toes leave the surface, marking the transition from the stance phase to the swing phase of the gait cycle. Maximum circumduction is measured as the maximal lateral (sideways) deviation of the swing leg from a straight line connecting the initial contact of one foot to the initial contact of the opposite foot during the swing phase of the gait cycle.

In addition, motor and sensory functions of the lower extremities were examined. These assessments were conducted for the clinical characterization of the patient group. A muscle function test [32] was used to test the muscle strength of the leg. The muscle strength was tested during the main movements in the hip, knee, and ankle joints of the affected side (hip flexion/extension, hip abduction/adduction, knee flexion/extension, dorsiflexion/plantar flexion). Six grades from 0 (no evidence of contractility) to 5 (normal muscle strength) are used. In the somatosensory test, patients were asked whether they felt a difference between touching the sole and the dorsum of the foot of the affected side compared to the less affected side. The less affected side was rated as 100%.

After the measurements, patients were asked about their impression regarding various aspects of the electrical stimulation. The questionnaire consisted of eight questions:

1. Did you have any concerns about treatment with this device?
2. Was the electrical stimulation pleasant?
3. Was wearing the device comfortable?
4. Did you feel pain during the electrical stimulation?
5. Did you feel pain after the training session?
6. Do you feel this electrical stimulation as being beneficial?
7. If possible, would you train with the device for a longer period of time?
8. Do you recommend this device for others?

A visual analogous scale ranging from 0 to 10 was used to quantify the patient's answers.

The CONSORT reporting guidelines was used in this article [33].

### Statistical analysis

Statistical analyses were performed using the IBM SPSS Statistics for Windows (Version 28.0.0.0 (190)). The data was first tested for normal distribution using the Shapiro–Wilk test. Differences in normally distributed parameters between with and without Incedo™ were detected using Student’s t-test for paired samples. Differences in non-normally distributed parameters between with and without Incedo™ were analyzed using Wilcoxon signed-rank test. The statistical analyses comparing participants with subacute and chronic stroke were conducted using the non-parametric Mann–Whitney U test. Correlations between numerical and ordinal data were assessed using Spearman’s correlation analysis. The level of significance for all tests was set at p < 0.05.

Degree of improvement. Finally, the percentage of change that occurred between with and without electrostimulation variables was calculated. For this purpose, the following equation was applied [7]:

$$\left|\mathrm{Degree\; of\; improvement}\right| =\frac{\left(\mathrm{Value\; with\; Incedo }-\mathrm{ Value\; without\; Incedo}\right)}{\mathrm{Value\; without\; Incedo}}\times100\mathrm{\%}$$

## Results

The 25 included patients completed all tests in the study. The descriptive data of the muscle function tests of the paretic leg and the somatosensory function tests of the foot are presented in Table 2.

**Table 2:** Results of the muscle function tests and somatosensory function tests

Category (affected side)	MV ± SD	Range (min.—max.)
Muscle strength in hip flexion (points: 0–5)	3.4 ± 0.7	1–4
Muscle strength in hip extension (points: 0–5)	3.7 ± 1.1	1–5
Muscle strength in hip abduction (points: 0–5)	3.2 ± 0.9	2–5
Muscle strength in hip adduction (points: 0–5)	3.5 ± 0.8	2–5
Muscle strength in knee flexion (points: 0–5)	3.2 ± 1.2	0–5
Muscle strength in knee extension (points: 0–5)	3.9 ± 0.9	2–5
Muscle strength in dorsiflexion (points: 0–5)	2.3 ± 1.3	0–4
Muscle strength in plantar flexion (points: 0–5)	2.5 ± 1.4	0–5
Sensitivity of the sole of the foot (%)	78.6 ± 24.3	20–100
Sensitivity of the dorsum of the foot (%)	78.6 ± 26.4	10–100

*MV* mean value, *SD* standard deviation, *min.* minimum, *max.* maximum

The muscle strength was reduced on the affected side, in particular for dorsiflexion and plantar flexion in the ankle joint followed by knee flexion, hip abduction and hip flexion. The patients showed an average sensitivity of almost 80% on both the dorsum and sole of the affected leg compared to the unaffected side.

17 patients were wearing a foot drop orthosis and 18 patients used assistive devices such as a cane or walker in everyday life. During the walk tests and the gait analysis, patients walked without an orthosis and without support from the therapist. However, 15 patients used a cane and 3 a walker throughout the testing.

On average the threshold value for Incedo™ was 8.6 mA ± 2.7 mA and the stimulus intensity during the tests was 30.2 mA ± 8.2 mA. Overall 11 patients showed a good response to stimulation of FR both during sitting and walking. Ten patients demonstrated a good response to stimulation of the FR while sitting but only moderate response while walking. One patient showed a good response to stimulation of the FR while sitting but only slight response while walking. One other patient presented a moderate response while sitting but only slight response while walking. Two patients showed a slight response to electrical stimulation of FR using Incedo™ while sitting and no response to the stimulation of the FR while walking.

**Fig. 2:**
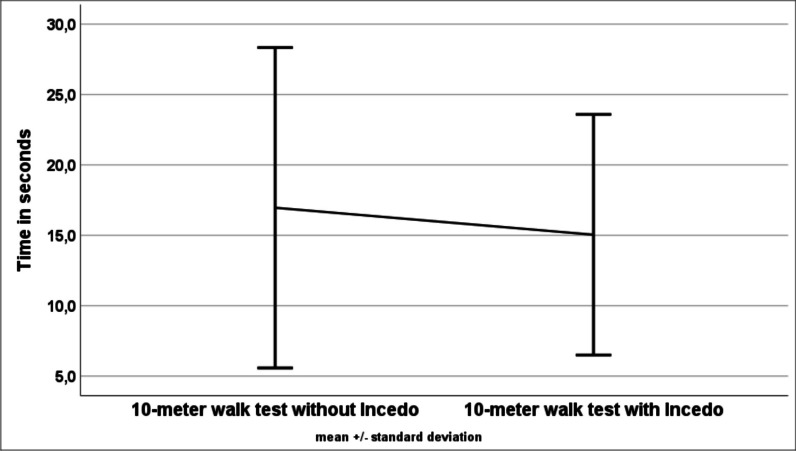
Results of the 10-m walk test (mean values and standard deviations) with and without Incedo™

**Fig. 3:**
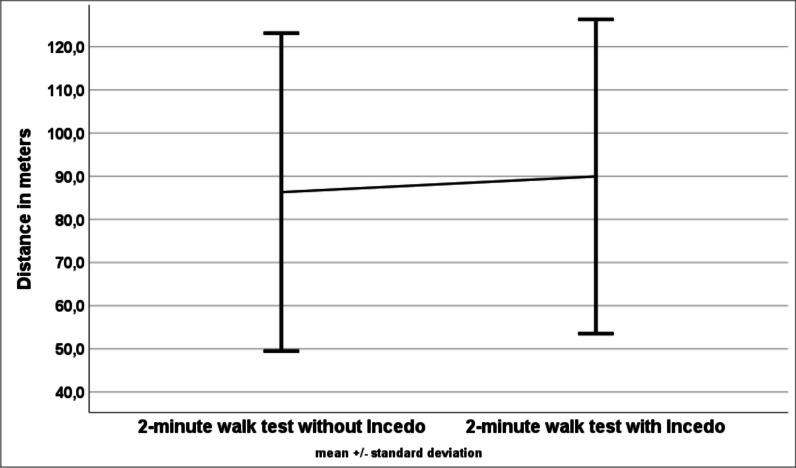
Results of the 2-min walk test (mean values and standard deviations) with and without Incedo™

Figure 2 presents the results for the 10mWT, and Fig. 3 shows the results for the 2minWT, both with and without Incedo™.

In the 10mWT, a significant difference was found between walking with and without Incedo™ (15.0 ± 8.5 s with Incedo™ versus 17.0 ± 11.4 s without Incedo™, p = 0.01). Patients walked on average 7.4% faster with electrostimulation compared to without electrostimulation. Nineteen patients improved their performance while using Incedo™. In one patient, the performance remained unchanged and five patients walked slower during electrical stimulation.

The 2minWT showed a significant change related to the Incedo™ use (90.0 ± 36.4 m with Incedo™ versus 86.3 ± 36.8 m without Incedo™, p = 0.03). Patients walked on average a 6.3% longer distance within two minutes with the Incedo™ system than without. Nineteen patients improved their performance while using Incedo™. In three patients no changes in the 2minWT could be observed during electrostimulation. In three other patients, the Incedo™ stimulation led to deterioration in performance.

Overall, 15 patients improved their performance in both functional tests while using Incedo™. Three patients showed improvement in one and remained unchanged in the other test. Five patients showed improved performance in one functional test but worse performance in the other. One patient's performance remained unchanged in one and decreased in the other test. Another patient showed a decrease in performance during electrostimulation in both tests.

The analysis showed that there was no correlation between the degree of response to electrostimulation while walking and the degree of improvement in 10mWT (*r* = 0.15, p = 0.5). However, there was a correlation between the degree of response to electrostimulation while walking and degree of improvement in 2minWT (*r* = 0.50, p = 0.01).

Participants with subacute and chronic stroke behaved very similarly. In both subgroups, performance improved when using the Incedo™. Neither the two values nor the degree of improvement in the 10mWT and the 2minWT were different between both groups (p > 0.30).

In the RehaGait system-based gait analysis, one parameter showed a significant difference during walking with and without Incedo™. Step length was significantly shorter while walking with electrostimulation than without it (0.77 ± 0.21 m versus 0.79 ± 0.21 m, p = 0.04). All other gait parameters (cadence, stance phase duration, swing phase duration, single support duration, double support duration, stride length, heel strike angle, toe off angle, maximum foot height and maximum circumduction) remained unchanged. Furthermore, a trend towards increasing the cadence while walking with Incedo™ was observed (83.4 versus 85.1 steps per minute, p = 0.09).

The subgroup gait analysis for participants with subacute and chronic stroke showed a significant difference in step length for participants with chronic stroke during walking with versus without Incedo™ (0.81 ± 0.20 m versus 0.84 ± 0.21 m, p = 0.047). All other parameters remained unchanged in these two groups (p > 0.08).

Table 3 presents the results of the patient questionnaire. All patients reported that they had no concerns about the treatment with Incedo™. They found the electrostimulation rather pleasant and definitely comfortable. Patients had no pain either during or after the treatment and if possible, they would exercise with the Incedo™ for a longer period of time. Furthermore, they considered the device to be effective and recommendable to others.

**Table 3:** The patient’s perspective: questionnaire answered by the patient

Question	Answer MV ± SD	Range (min.–max.)
Did you have any concerns about treatment with this device?	0.3 ± 1.0	0–4.5
Was the electrical stimulation pleasant?	5.9 ± 2.2	2.0–10
Was wearing the device comfortable?	9.5 ± 1.2	4.3–10
Did you feel pain during the electrical stimulation?	0.5 ± 1.6	0.0–6.8
Did you feel pain after the training session?	0.0 ± 0.0	0.0–0.0
Do you feel this electrical stimulation as being beneficial?	8.1 ± 2.5	0.0–10
If possible, would you train with the device for a longer period of time?	8.2 ± 2.6	0.0–10
Do you recommend this device for others?	8.8 ± 2.4	0.0–10

Answers were given on a visual analogous scale ranging from “0” corresponding to “not at all” to “10” corresponding to “yes, full agreement”

*MV* mean value, *SD* standard deviation, *min*. minimum, *max.* maximum

## Discussion

This study showed that stroke patients walked significantly faster in 10mWT and a longer distance in 2minWT with Incedo™ compared to without electrostimulation. Thus, our first hypothesis that gait performance can be improved even within a single session of FR stimulation is supported by the results. Data analysis in greater detail showed that 23 of 25 patients improved their performance in at least one functional test. Only two patients failed to benefit from a single session of FR stimulation. In contrast to other patients, these two patients showed only a slight response to electrical stimulation of FR using Incedo™ while sitting and no visible response to the stimulation of the FR while walking. Furthermore, the FR while walking could only be moderately triggered by Incedo™ in some patients. There were several reasons for this. Five patients could not tolerate a further increase of stimulation intensity which prohibited a stronger response to electrostimulation. Either they found the higher stimulation intensity uncomfortable and/or disturbing or the higher stimulation intensity triggered spasticity in the affected leg. Two other patients became accustomed very quickly to the higher stimulation intensity during walking so that the initially good response to FR stimulation had diminished within 15 min. There were also two patients for whom it was not clear why they showed moderate and good response while sitting and only slight response while walking despite an increase in stimulation intensity. In summary, the inconsistent responses to FR stimulation can be attributed to the inherent variability in how individuals react to such interventions, as supported by our data. This variability in response may depend on factors unique to each patient, contributing to the mixed outcomes observed in our study.

The positive correlation between the degree of response to electrostimulation while walking and degree of improvement in 2minWT confirms the importance of responsiveness to FR stimulation for patient performance.

While the current results do not offer substantial support for the second hypothesis, suggesting limited changes in gait parameters within a single session of FR stimulation, it is crucial to note that only one parameter—step length—showed a significant difference between walking with and without Incedo™, specifically, a reduction of two centimeters during electrostimulation. This alteration in step length represents a subtle effect. While it is likely that a majority of patients exhibited this discreet reduction in step length for the difference to reach statistical significance, the magnitude of the average shortening (2% of the mean step length) does not appear to hold clinical relevance. We speculate that increased hip flexion led to the foot being raised more vertically (rather than advanced forward), resulting in a comparatively shorter step length. However, alterations in the gait pattern during walking with Incedo™ were visually evident in the majority of patients. In particular, increased foot dorsiflexion and hip flexion were noticeable as patients walked with electrostimulation. The reasons why the gait analysis system RehaGait failed to capture these changes remain speculative and unclear. It is possible that utilizing motion analysis with multiple sensors placed on different body segments might be more suitable for accurately capturing the visually observed changes in gait patterns. In the intervention studies mentioned in the "Background" section [19–22], there was no examination of the isolated effect of FR stimulation on the patients' gait parameters. Instead, these studies investigated the impact of the combination of gait training and FR electrostimulation on gait performance and parameters. As a result, there are no comparable data available for this specific context. The only exception is a single-case study involving a severely affected, chronic stroke patient in whom the effect of activating the FR on the patient's gait pattern was examined simultaneously [22]. In this case, a longer stride length, a shorter double support time, a slightly longer duration of the stance phase, and a shorter duration of the swing phase during FR activation were reported.

This study demonstrates, for the first time, the effects of FR stimulation in chronic stroke patients. Our results indicate that chronic stroke patients benefit from the stimulation to a similar degree as subacute stroke patients do. Thus, time after a stroke does not affect the efficacy of Incedo™ on gait performance and gait parameters.

The patients rated the device as effective and recommendable to others. Most patients would exercise with Incedo™ for a longer period of time, thus suggesting an overall good acceptance of the device. The electrical stimulation was not found pleasant in all patients. Nevertheless, the patients rated the FR stimulation as being positive.

A major question is whether the improvement of gait performance observed in this study is not only statistically significant but also clinically relevant. In literature, improvements of 0.13 m/s [34], 0.16 m/s [35] or 0.175 m/s [36] are considered to be clinically meaningful. In our patient group, gait velocity as measured during the 10mWT improved by 0.08 m/s during FR stimulation. This value is clearly below the one proposed in the literature. A further analysis indicated that seven patients improved their gait velocity by 0.13 m/s, thus meeting the criterion of a clinically relevant change. These seven patients exhibited the following characteristics: the average age was 59.8 years, with a gender distribution of 5 males and 2 females. Among them, 2 were participants with subacute stroke and 5 were participants with chronic stroke. Additionally, in terms of orthotic use, 5 of these patients regularly used an Ankle–Foot Orthosis (AFO) in their daily life, while 2 did not. However, it should be kept in mind that FR stimulation with the Incedo™ is meant as a tool for exercising for a longer period of time. This study was designed to examine the effect of FR stimulation during walking within a single session. Thus, over time, gait improvements might become larger. This needs to be examined in a controlled therapeutical trial, because there is still limited evidence for participants with subacute stroke and no evidence for participants with chronic stroke. Furthermore, it should be investigated who benefits most from this type of therapy. E.g Spaich et al. (2014) reported the largest therapeutical gains of FR activation in patients with the most impaired mobility [21]. The question of the optimal therapeutic dose should also be addressed in future studies.

## Limitations/Lessons learnt from this study

Our study results provide some evidence that the inclusion criteria should additionally include the requirement of a visible response to FR stimulation during walking. We observed that patients need less stimulation intensity to trigger the FR while sitting than while walking. Furthermore, some patients need more than one 30 min session to get used to the higher stimulation intensity during walking. In addition, the exclusion criteria should also be adjusted. Patients who adjust to the electrical stimulus within 30 min or patients in whom a higher stimulation intensity triggers spasticity in the affected leg should be excluded from the study.

In the context of this study, the implementation of a sham stimulation proved unfeasible due to the nature of electrical stimulation targeting the FR. Even with subthreshold stimulation, the afferent pathways are activated, potentially influencing motor responses. The activation of afferent pathways—even at levels beneath the motor threshold—can induce subtle changes in motor responses, making the establishment of an indistinguishable sham stimulation a challenging feat [37, 38]. The intricate relationship between stimulation and neurophysiological responses further complicates the feasibility of sham interventions, particularly in studies involving nuanced sensory-motor pathways such as those targeted in our investigation [39–41]. This design would be more appropriate for studying a dose–response relationship. If the stimulus intensity is above threshold for one or very few electrical stimuli and then turned down to zero, the patient will easily recognize the difference and will, therefore, easily detect whether he/she is currently in the “real stimulation “ or the “sham stimulation“ condition.

As a limitation of our study, it should be noted that the activation of FR stimulation during walking did not benefit all participants. Specifically, our findings indicate that a significant proportion, 44% or 20 out of 45 enrolled participants, may not have responded to this form of stimulation.

A further limiting aspect in this study arises from the absence of blinding for both the participants and the assessors concerning the treatment. The experimental setup did not allow such a blinding due to the fact that all patients experienced the stimulation and, visually, the reaction to the stimulation could be observed in all patients while sitting. Additionally, a response to the stimulation of the FR during walking was observable in most patients.

The qualitative approach employed in assessing the FR response via visual categorization into four distinct classifications is acknowledged as a limitation due to its inherent subjectivity. The assessment, categorized as good, moderate, slight, or none, was reliant on visual observation by investigators, which might lack the rigor and objectivity desired in research evaluation methodologies. A more quantitative and objective assessment, such as employing the Inertial Measurement Unit (IMU) system, can provide a more precise and standardized measurement of the stimulation response. In future studies, we aim to overcome this limitation by integrating more objective measurement instruments, particularly by employing quantitative assessment tools like the IMU system.

In this study, we had decided to conduct only a maximum walking speed but not a comfortable walking speed of the 10mWT in order to prevent patient fatigue since some patients had low physical endurance. An option for future studies could be performing both tests for more resilient patients and conducting only one of the assessments for less resilient patients to address this issue.

The measurement of sensitivity in this study has a subjective nature and is intended to be complemented in future studies by more robust and objective measurements.

## Conclusions

This study comprehensively investigated the immediate effects of FR stimulation on gait performance and parameters in participants with subacute and chronic stroke. Our findings confirm that FR stimulation, even in a single session, can significantly improve walking speed and distance in the 10-m and 2-min walk tests. While the majority of gait parameters remained unchanged, a slight reduction in step length was observed.

The observed improvements in walking speed and distance, though statistically significant, warrant further investigation to assess their clinical relevance and long-term sustainability. Considering the mixed responses to FR stimulation, future research should focus on identifying patient-specific factors that influence responsiveness to this therapy. Additionally, therapeutic trials are needed to establish the optimal duration and intensity of FR stimulation for maximal therapeutic benefit.

In summary, our study provides preliminary evidence supporting the use of FR stimulation in post-stroke gait rehabilitation. The positive patient feedback and the absence of adverse effects further reinforce the potential of Incedo™ as a valuable tool in neurorehabilitation. Future research should aim to build upon these findings, exploring the long-term effects of FR stimulation and refining patient selection criteria to maximize therapeutic outcomes.

### Supplementary Information

Supplementary Material 1.

## Data Availability

All relevant data are within the paper.

## Abbreviations

*AFO*: Ankle–Foot Orthosis
*FR*: Flexor reflex
*max.*: Maximum
*IMU*: Measurement Unit
*MV*: Mean value
*m*: Meters
*mA*: Milliampere
*min.*: Minimum
*n*: Number of patients
*s*: Seconds
*SD*: Standard deviation
*10mWT*: 10-Meter walk test
*2minWT*: 2-Minute walk test
