# Supplementary material for: Effects of flexor reflex stimulation on gait aspects in stroke patients: randomized clinical trial
Source: J Neuroeng Rehabil. 2024 May 28;21:83. doi: 10.1186/s12984-024-01377-y (PMC11131186; doi:10.1186/s12984-024-01377-y)
Supplement: Supplementary file 1 — Supplementary Material 1. [file 12984_2024_1377_MOESM1_ESM.docx]

**Supplementary materials**

Descriptive statistics for participants with subacute and chronic stroke for all gait parameters with and without Incedo^TM^

| **Gait parameters** | **Number of patients** | **Mean** | **Standard deviation** |
| --- | --- | --- | --- |
| Stride duration without Incedo^TM^ (s) | 25 | 1.50 | .37 |
| Stride duration with Incedo^TM^ (s) | 25 | 1.47 | .35 |
| Stride length without Incedo^TM^ (m) | 25 | .79 | .21 |
| Stride length with Incedo^TM^ (m) | 25 | .77 | .21 |
| Cadence without Incedo^TM^ (number of single steps per minute) | 25 | 83.4 | 17.07 |
| Cadence with Incedo^TM^ (number of single steps per minute) | 25 | 85.1 | 17.6 |
| Stand phase duration without Incedo^TM^ affected side (%) | 25 | 68.3 | 8.03 |
| Stand phase duration with Incedo^TM^ affected side (%) | 25 | 66.6 | 6.9 |
| Stand phase duration without Incedo^TM^ less affected side (%) | 25 | 72.4 | 6,3 |
| Stand phase duration with Incedo^TM^ less affected side (%) | 25 | 72.8 | 7.06 |
| Swing phase duration without Incedo^TM^ affected side (%) | 25 | 31.6 | 7.99 |
| Swing phase duration with Incedo^TM^ affected side (%) | 25 | 33.4 | 6,9 |
| Swing phase duration without Incedo^TM^ less affected side | 25 | 27.6 | 6.3 |
| Swing phase duration with Incedo^TM^ less affected side (%) | 25 | 27.2 | 7.06 |
| Single support duration without Incedo^TM^ affected side (%) | 25 | 27.7 | 6.1 |
| Single support duration with Incedo^TM^ affected side (%) | 25 | 27.1 | 7.02 |
| Single support duration without Incedo^TM^ less affected side | 25 | 31.1 | 7.8 |
| Single support duration with Incedo^TM^ less affected side (%) | 25 | 33.3 | 6.8 |
| Double support duration without Incedo^TM^ affected side (%) | 25 | 28.4 | 15.9 |
| Double support duration with Incedo^TM^ affected side (%) | 25 | 29.6 | 14.7 |
| Double support duration without Incedo^TM^ less affected side (%) | 25 | 26.2 | 17.4 |
| Double support duration with Incedo^TM^ less affected side (%) | 25 | 24.4 | 17.9 |
| Heel strike angle without Incedo^TM^ affected side (°) | 25 | 5.99 | 5.6 |
| Heel strike angle with Incedo^TM^ affected side (°) | 25 | 5.60 | 4,7 |
| Heel strike angle without Incedo^TM^ less affected side (°) | 25 | 10.22 | 8.9 |
| Heel strike angle with Incedo^TM^ less affected side (°) | 25 | 10.90 | 9.5 |
| Toe off angle without Incedo^TM^ affected side (°) | 25 | -11.9 | 23.9 |
| Toe off angle with Incedo^TM^ affected side (°) | 25 | -9.0 | 24.6 |
| Toe off angle without Incedo^TM^ less affected side (°) | 25 | -17.9 | 36.6 |
| Toe off angle with Incedo^TM^ less affected side (°) | 25 | -19.7 | 34.4 |
| Maximum foot height without Incedo^TM^ affected side (m) | 25 | .06 | .02 |
| Maximum foot height with Incedo^TM^ affected side (m) | 25 | .06 | .02 |
| Maximum foot height without Incedo^TM^ less affected side (m) | 25 | .05 | .013 |
| Maximum foot height with Incedo^TM^ less affected side (m) | 25 | .05 | .015 |
| Maximum circumduction without Incedo^TM^ affected side (m) | 25 | .067 | .041 |
| Maximum circumduction with Incedo^TM^ affected side (m) | 25 | .062 | .041 |
| Maximum circumduction without Incedo^TM^ less affected side (m) | 25 | .024 | .01 |
| Maximum circumduction with Incedo^TM^ less affected side (m) | 25 | .023 | .01 |

*s* seconds, *m* meters, *%* percent, *s* seconds, *°* Angle
